# Identification and validation of PCSK9 as a prognostic and immune-related influencing factor in tumorigenesis: a pan-cancer analysis

**DOI:** 10.3389/fonc.2023.1134063

**Published:** 2023-10-04

**Authors:** Chao Sun, Guoji Zhu, Conghuan Shen, Shungen Huang, Ruidong Li, Jianhua Li, Zhenyu Ma, Zhengxin Wang

**Affiliations:** ^1^ Department of General Surgery, Huashan Hospital, Fudan University, Shanghai, China; ^2^ Surgery Intensive Care Unit, Children’s Hospital of Suzhou University, Suzhou, China; ^3^ Department of General Surgery, Children’s Hospital of Suzhou University, Suzhou, China

**Keywords:** pcsk9, tumorigenesis, prognosis, immune infiltrate, pan-cancer

## Abstract

**Introduction:**

Proprotein convertase subtilisin/kexin-9 (PCSK9) has been primarily studied in the cardiovascular field however, its role in cancer pathophysiology remains incompletely defined. Recently, a pivotal role for PCSK9 in cancer immunotherapy was proposed based on the finding that PCSK9 inhibition was associated with enhancing the antigen presentation efficacy of target programmed cell death-1 (PD-1). Herein, we provide results of a comprehensive pan-cancer analysis of PCSK9 that assessed its prognostic and immunological functions in cancer.

**Methods:**

Using a variety of available online cancer-related databases including TIMER, cBioPortal, and GEPIA, we identified the abnormal expression of *PCSK9* and its potential clinical associations in diverse cancer types including liver, brain and lung. We also validated its role in progression-free survival (PFS) and immune infiltration in neuroblastoma.

**Results:**

Overall, the pan-cancer survival analysis revealed an association between dysregulated PCSK9 and poor clinical outcomes in various cancer types. Specifically, PCSK9 was extensively genetically altered across most cancer types and was consistently found in different tumor types and substages when compared with adjacent normal tissues. Thus, aberrant DNA methylation may be responsible for PCSK9 expression in many cancer types. Focusing on liver hepatocellular carcinoma (LIHC), we found that PCSK9 expression correlated with clinicopathological characteristics following stratified prognostic analyses. PCSK9 expression was significantly associated with immune infiltrate since specific markers of CD8+ T cells, macrophage polarization, and exhausted T cells exhibited different PCSK9-related immune infiltration patterns in LIHC and lung squamous cell carcinoma. In addition, PCSK9 was connected with resistance of drugs such as erlotinib and docetaxel. Finally, we validated PCSK9 expression in clinical neuroblastoma samples and concluded that PCSK9 appeared to correlate with a poor PFS and natural killer cell infiltration in neuroblastoma patients.

**Conclusion:**

*PCSK9* could serve as a robust prognostic pan-cancer biomarker given its correlation with immune infiltrates in different cancer types, thus potentially highlighting a new direction for targeted clinical therapy of cancers.

## 1 Introduction

The complex process of tumorigenesis involves interactions between the immune system and tumor. Currently, targeting cytotoxic T lymphocyte-associated protein 4 (CTLA-4) and PD-1 has provided superior anticancer effects in colorectal and lung cancer compared to conventional chemotherapy (1). However, most cancer patients continue to suffer poor outcomes from immunotherapies with observed overall objective response rates approximately 15–25% in various cancer types (2). Recently, the 3rd generation immune checkpoint blockades have received widespread attention, because of their combined immunotherapy strategies in the anti-tumor microenvironment (TME) (3, 4). In order to maximize the synergistic benefits in strengthening the immune response, it is vital to verify and highlight novel immune-related therapeutic targets in malignancies (5). Performing a pan-cancer investigation of putative genes could help determine its involvement in clinical prognosis and immunological functions due to the intricate relationship between carcinogenesis and TME.

In 2003, human *PCSK9* gene mutation was identified as the third genetic cause of autosomal dominant familial hypercholesterolemia for the first time (6). Low-density lipoprotein cholesterol (LDL-C) levels were shown to increase in response to PCSK9-mediated degradation of low-density lipoprotein cholesterol receptors (LDLR) (7, 8). Clinical research has finally confirmed the key role for PCSK9 in cholesterol metabolism, because the inhibition of LDL-C mediated by PCSK9 has expanded the therapeutic tools that can be used to treat individuals with residual LDL-C related cardiovascular risk (9). In addition to regulating cholesterol metabolism, *in vitro* and *in vivo* studies have also found that PCSK9 is involved in various other physiological processes (10). For example, inhibition of PCSK9 was demonstrated to reduced myocardial ischemia-/reperfusion injury via BNIP-3 mediated autophagic pathway and improved myocardial infarct size and subsequent cardiac function. By investigating the functional role of PCSK9 inhibitions in the metabolic targets, it could potentially lead to better understanding of the pathogenesis of myocardial infarction and ischemic stroke, as well as provide a potential therapeutic target for its management (11). Additionally, the knowledge gained from exploring the role of PCSK9 in cell proliferation and apoptosis could help to elucidate the potential involvement of this protein in cancer risk, providing insight into potential preventive strategies (12). A recent study showed that inhibiting PCSK9 enhanced the antigen presentation efficacy of PD-1 and influenced the tumor response to immune checkpoint treatment, although through a mechanism unrelated to its role in controlling cholesterol (13, 14). This novel finding highlighted that PCSK9 inhibition was a potential strategy for improving immune checkpoint treatment for cancer. Given its likely engagement with PD-1 in tumor immunotherapy, PCSK9 might provide useful insight into tumor development and immune treatment response. Therefore, it is vital to comprehensively assess PCSK9 clinical prognostic association with tumors.

In the current study, we thoroughly examined the relationships among *PCSK9* expression, methylation, mutation, and patient prognosis in 33 different cancer types. In order to further examine aberrant patterns and the possible clinical importance of PCSK9 across various cancer types, a survival association study was carried out. We also investigated the relationship between *PCSK9* expression and immunological checkpoints and six tumor-infiltrating immune cells in 33 TME. *PCSK9* expression changes in neuroblastoma was not verified in the databases used in this study. Therefore, we further verified the expression changes of *PCSK9* in neuroblastoma clinical samples as a supplemental study. Our results emphasize the potential relevance of PCSK9 across malignancies and indicated that it may be a predictive biomarker associated with immune infiltration in various tumors.

## 2 Materials and methods

### 2.1 Gene expression analysis

#### 2.1.1 Tumor immune estimation resource database

In this study, *PCSK9* mRNA expression levels in different tumor disease tissues and normal tissues were retrieved from the TIMER database (15). Thirty-three cancer types were included: adrenocortical carcinoma (ACC), bladder urothelial carcinoma (BLCA), breast invasive carcinoma (BRCA), colon adenocarcinoma (COAD), lymphoid neoplasm diffuse large B-cell lymphoma (DLBC), esophageal carcinoma (ESCA), glioblastoma multiforme (GBM), head and neck squamous cell carcinoma (HNSC), kidney chromophobe (KICH), kidney renal clear carcinoma (KIRC), kidney renal papillary cell carcinoma (KIRP), acute myeloid leukemia (LAML), brain lower grade glioma (LGG), liver hepatocellular carcinoma (LIHC), lung adenocarcinoma (LUAD), lung squamous cell carcinoma (LUSC), ovarian serous cystadenocarcinoma (OV), pancreatic adenocarcinoma (PAAD), prostate adenocarcinoma (PRAD), rectum adenocarcinoma (READ), skin cutaneous melanoma (SKCM), stomach adenocarcinoma (STAD), testicular germ cell tumors (TGCT), thyroid carcinoma (THCA), thymoma (THYM), uterine corpus endometrial carcinoma (UCEC), cervical squamous cell carcinoma (CESC), cholangiocarcinoma (CHOL), mesothelioma (MESO), pheochromocytoma and paraganglioma (PCPG), sarcoma (SARC), uveal melanoma (UVM), and uterine carcinosarcoma (UCS).

#### 2.1.2 Gene expression profiling interactive analysis 2 database

The GEPIA2 database (http://gepia2.cancer-pku.cn/) was used to analyze *PCSK9* expression profiles between disease tumors and normal tissues (16). We also explored the distribution of *PCSK9* in specific tumor stages and drew a violin diagram of tumor stages. The distribution of *PCSK9* in different cancers and the clinical stage of the tumors were initially explored, and a violin map of the tumor stage was drawn.

#### 2.1.3 TISIDB portal

TISIDB is a website for gene- and tumor-immune interaction (http://cis.hku.hk/TISIDB/index.php/) (17). It was used to analyze *PCSK9* gene expression in different immune subtypes, including C1 (wound healing), C2 (IFN-γ dominant), C3 (inflammatory), C4 (lymphocyte depleted), C5 (immunologically quiet), and C6 (TGF-β dominant) subtypes. *PCSK9* gene expression was also analyzed in different molecular subtypes of tumor samples from The Cancer Genome Atlas (TCGA).

### 2.2 Genetic alteration analysis

#### 2.2.1 cBioPortal database

The mutation levels of the *PCSK9* gene were obtained from the online cBioPortal database (https://www.cbioportal.org/). We searched in the “mutation” module of the website to obtain the specific mutation site information on the *PCSK9* functional and structural domain map (18, 19).

#### 2.2.2 UALCAN network

DNA methyltransferase alteration plays a vital role in chromatin structure and gene expression levels. The UALCAN network (http://ualcan.path.uab.edu) was used to analyze differential DNA methylation of *PCSK9* between tumor and normal tissues (20).

#### 2.2.3 GSCALite platform

The GSCALite platform (http://bioinfo.life.hust.edu.cn/web/GSCALite/) was selected to obtain the copy number variations (CNV) of *PCSK9* between cancer tissues and adjacent tissues in 33 types of cancers in the TCGA (21).

### 2.3 Gene set enrichment analysis of PCSK9

Here, ssGSEA is used to calculate the enrichment score of each sample and obtain the correlation between *PCSK9* expression and pathway score. R software GSVA package was used to analyze, choosing parameter as method = ‘ssgsea’. The correlation between genes and pathway scores was analyzed by Spearman correlation. ClusterProfiler package (version: 3.18.0) in R software was employed to analyze the Gene Ontology (GO) function of potential targets and enrich the Kyoto Encyclopedia of Genes and Genomes (KEGG) pathway, p <0.05 or FDR <0.05 is considered to be a meaningful pathway (enrichment score with −log10 (P) of more than 1.3).

### 2.4 Stemness analysis of PCSK9

To analyze the stemness features, we performed the spearman correlation between mRNAsi of various tumors and *PCSK9* expression. This method refers to the OCLR algorithm constructed by Malta, which contains 11774 different gene profiles (22).

### 2.5 Survival prognosis analysis

#### 2.5.1 PrognoScan database

The correlation of *PCSK9* expression with pan-cancer survival was analyzed using PrognoScan (23). Specifically, *PCSK9* expression levels were searched in all available microarray datasets in PrognoScan to determine its association with prognosis, including overall survival (OS) and disease-free survival (DFS). The threshold was set at a Cox *p* value < 0.05. We explored the impact of *PCSK9* expression on OS and DFS in each cancer type.

#### 2.5.2 Kaplan-Meier Plotter database

Kaplan-Meier Plotter (https://kmplot.com/analysis/) is an online database containing gene expression and clinical information for 54,000 samples on 21 cancer types (24). To assess the clinical prognostic value of specific genes, patient samples were divided into two groups according to median gene expression (high vs. low expression). Kaplan-Meier survival curves were used to analyze pan-cancer OS rates. The association between *PCSK9* expression and OS in different tumor tissues was analyzed, and the 95% confidence interval (95% CI) and the hazard ratio (HR) of the log-rank *p* values were calculated. Furthermore, we analyzed the relationship between *PCSK9* expression in LIHC and OS and recurrence-free survival (RFS) and calculated log-rank *p* values and 95% confidence intervals (CIs) for risk ratios (HRs). The impact of various risk factors on tumor prognosis was also analyzed to explore the impact of different clinical characteristics on tumor prognosis.

#### 2.5.3 GEPIA database

The survival analysis module in the GEPIA database was used to obtain the OS and DFS data of patients with different *PCSK9* expression across TCGA and Genotype-Tissue Expression Project (GTEx). The survival module was also used to explore the expression patterns between *PCSK9* and survival factors in tumor patients (16).

### 2.6 Immune correlation analysis

#### 2.6.1 TIMER database

##### 2.6.1.1 Immune infiltrating cells

To focus on the role of immune cells in the TME, the expression profile data of tumor samples in TCGA were analyzed using the “immune gene” module of the TIMER 2.0 database. The xCell algorithms were used to explore the potential relationship between the level of cancer-related immune cell infiltration and *PCSK9* gene expression in different cancer types found in TCGA. The tumor-infiltrating immune cells were correlated with gene expression to assess the level of immune cell infiltration in the TME.

##### 2.6.1.2 Immune checkpoint

Over 40 common immune checkpoint genes were identified, and the correlation between *PCSK9* and the immune checkpoint genes was analyzed using the R software package in the SangerBox database (http://www.sangerbox.com/) and presented in a heat map.

##### 2.6.2.3 Immune infiltration

TIMER is an ideal database for systematically analyzing immune infiltration in multiple cancer types. We analyzed the relationship between *PCSK9* expression and immune infiltrating cells, including B cells, CD4+ T cells, CD8+ T cells, neutrophils, macrophages, and dendritic cells. ESTIMATE is a tool for predicting tumor purity and stromal and immune cell infiltration into tumor tissue using gene expression data. The ESTIMATE algorithm was used to infer the immune score of each sample (25).

##### 2.6.2.4 Copy number alterations and immune infiltration

The correlation between different somatic copy number alterations (SCNAs) and immune cell infiltration affecting *PCSK9* expression was also explored by using TIMER. Four types of alterations (arm-level deletion, diploid/ordinary, arm-level gain, high amplification) were analyzed and compared in the SCNAs. The infiltration level of each SCNA category was compared with that of normal tissue using a two-sided Wilcoxon rank-sum test.

### 2.7 Drug sensitivity analysis

The Drug Sensitivity Genomics Project (GDSC) and The Cancer Therapeutics Response Portal (CTRP)are two databases, which combine drug sensitivity and genome data sets to promote the new therapeutic biomarkers for cancer therapy. Through these two databases, we investigated the role of *PCSK9* expression in cancer therapeutic response. Pearson correlation analysis was performed to obtain the correlation between *PCSK9* mRNA expression and drug IC50. P-value was adjusted by FDR.

### 2.8 Neuroblastoma specimen collection

A total of 25 neuroblastoma (NB) patients from Children’s Hospital of Soochow University (Suzhou, China) were consecutively enrolled in a study between January 2016 and December 2019 and followed up until December 2021. NB specimens and adjacent normal tissues were collected at the time, frozen in liquid nitrogen, and stored at −80^°^C until use. No patients received chemotherapy or radiotherapy or any treatment for the tumor before surgery or tissue biopsy. The sample collection and related experiments met the ethical requirements of the Children’s Hospital of Suzhou University, Suzhou, China. The Clinical Research Ethics Committee approved this study at the Children’s Hospital of Suzhou University, Suzhou, China, and all patients or their parents signed informed consent forms.

### 2.9 Western blot assay and quantitative real-time PCR

Proteins were isolated from tissues using RIPA lysis buffer (Biotime, Shanghai, China), separated using SDS-PAGE, and transferred onto a nitrocellulose membrane (Bio-Rad Laboratories, CA, USA). Then, we blocked proteins with 5% skim milk for 30 min and incubated the membranes with diluted primary antibodies. Primary antibodies for GAPDH (ab8245, 1:10000 dilution; Abam, Cambridge, UK), PCSK9 (ab181142, 1:1000 dilution; Abam, Cambridge, UK), CD11b (ab133357, 1:1000 dilution; Abam, Cambridge, UK), CD45 (ab40763, 1:5000 dilution; Abam, Cambridge, UK), CD68 (ab213363, 1:1000 dilution; Abam, Cambridge, UK), and BSA-1 (ab219724, Abam, Cambridge, UK) were purchased from Abcam (Cambridge, UK). After incubating with horseradish peroxidase-conjugated secondary antibodies, the immune complexes were detected with an ECL detection kit (Millipore, Billerica, MA, USA) and quantified using a Gel-Pro Analyzer (Media Cybernetics Corporation, USA). Total RNA was isolated from patients with NB by using Trizol and then converted into cDNA by reverse transcription (GoScriptTM Reverse Transcription system, USA). RT-qPCR was used to assay the mRNA expression of *PCSK9*, immune-related genes, and pathway-related genes. *PCSK9* primers were purchased from Sino Biological Inc. The primers used for qRT-PCR were as follows: *PCSK9*, 5′-GCT GAGCTGCTCCAGTTTCT-3′ (forward) and 5′-AAT GGCGTAGACACCCTCAC-3′ (reverse); and *GAPDH*, 5′-AAGGTGAAGGTCGGAGTCAAC-3′ (forward) and 5′-GGGGTCATTGATGGCAACAATA-3′ (reverse).

### 2.10 Statistical analysis

Gene expression data from the TCGA and GTEx databases were analyzed by using t-tests. The Kruskal-Wallis test was used to evaluate the difference among various tissues, and the Wilcoxon test was used to determine the gene expression differences between normal and tumor tissues. In PrognoScan, univariate Cox regression analysis was used to analyze the survival time of patients with the HR and *p* value. In GEPIA and Kaplan-Meier Plotter, log rank test was used to compare survival rate of patients stratified according to the different expression levels of *PCSK9*. Other online analysis websites of GEPIA2, cBioportal and GSCALite were also used. Spearman correlation analysis was calculated between the expression of *PCSK9* and the level of infiltrating immune cells, the level of immunosuppressant or immunostimulant factors and infiltration scores of six immune infiltrations. Correlations were considered statistically significant when *p* < 0.05 for all statistical analyses. The experimental data were analyzed using SAS 9.3 statistical software. Statistical analysis was performed using the *t* test. Differences were considered statistically significant at *p* < 0.05. OS was defined as the time interval between the date of surgery and the date of progression or death. Survival analyses were conducted by Kaplan–Meier curves (*p* values from log-rank test) by R version 3.5.3 and the survival package. HRs were calculated using the R package.

## 3 Results

### 3.1 mRNA expression level of *PCSK9* in human cancer

**Figure 1:**
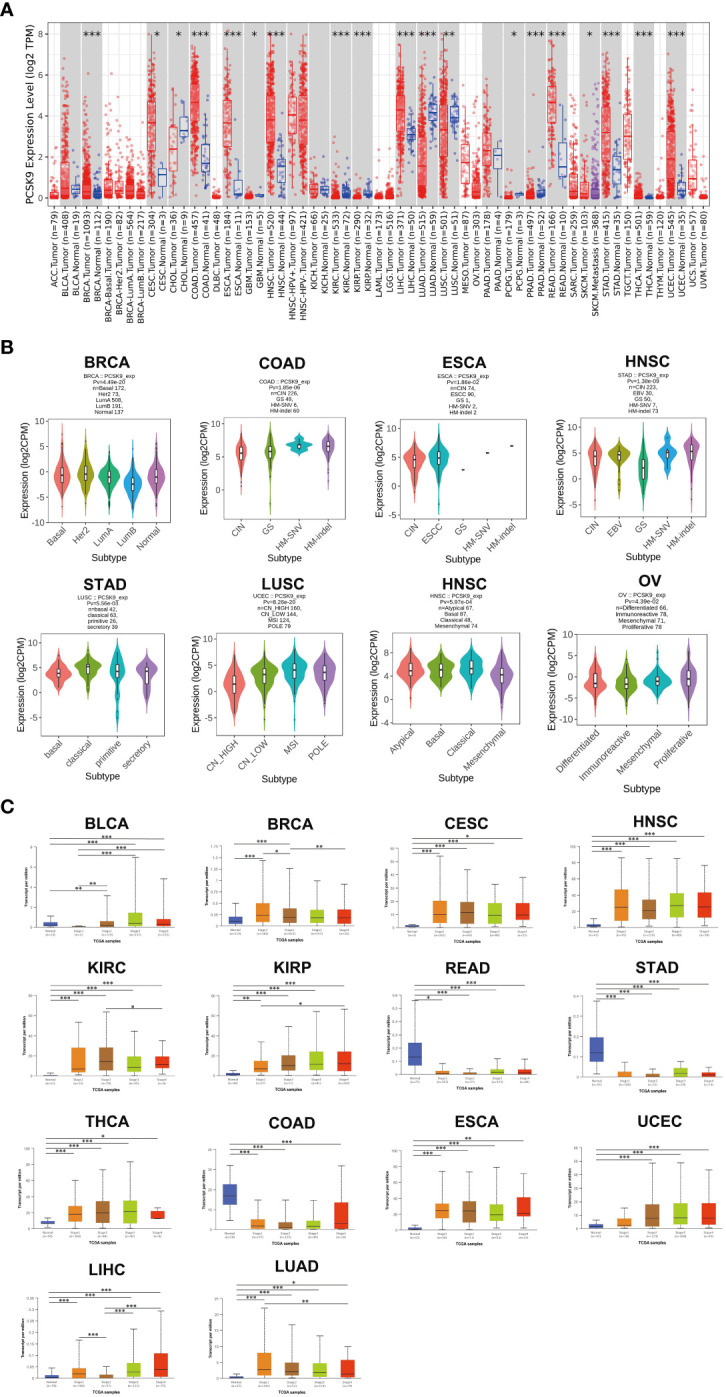
**(A)** PCSK9 expression levels in different cancer types. Increased or decreased expression of *PCSK9* compared with normal tissues across different cancer types from the TCGA database in TIMER (*p < 0.05, **p < 0.01, ***p < 0.001). **(B)** Pan-cancer *PCSK9* expression in different subtypes. (A–H), Pan-cancer differential expression of *PCSK9* in different cancer subtypes in the indicated tumor types from GEPIA. **(C)** Correlation between *PCSK9* expression and the main pathological WHO stages for BLCA, BRCA, CESC, HNSC, KIRC, KIRP, READ, STAD, THCA, COAD, ESCA, UCEC, LIHC, and LIHC (A–N) from the UALCAN database (*p < 0.05, **p < 0.01, ***p < 0.001).

Abnormal *PCSK9* expression has been reported in various cancer types. Previous studies on *PCSK9* expression in cancer used several research methods, such as DNA microarrays, but were limited to relatively small sample sizes and limited numbers of cancer types. This study has provided a more comprehensive analysis of *PCSK9* expression in cancer. Since *PCSK9* has a potential role as an important new target for cancer diagnosis and prognosis, we analyzed the *PCSK9* mRNA levels across different cancers from TIMER, GEPIA, and UALCAN databases. Data from these databases indicated that *PCSK9* mRNA expression had inter-tumor heterogeneity, with some tumors having very high levels of *PCSK9* (BRCA, CESC, CHOL, COAD, ESCA, HNSC, LIHC, READ, SKCM, STAD, THCA, and UCEC). In contrast, others were characterized by low levels of *PCSK9* expression (LUAD, PRAD, KIRP, KIRC, PCPG, LUSC, and GBM) (**Figure 1A**). Different stages and subtypes of a tumor may exhibit differential expression of *PCSK9*. Thus, we further assessed *PCSK9* expression in different clinical stages and subtypes from GEPIA and UALCAN. Briefly, differential expression of *PCSK9* was obtained from GEPIA to correlate with clinical subtypes of tumors, including BRCA, COAD, ESCA, HNSC, STAD, LUSC, OV, and UCEC (**Figure 1B**). As shown in **Figure 1C**, *PCSK9* expression was elevated in some tumors, including BLCA, BRCA, CESC, HNSC, READ, STAD, THCA, COAD, ESCA, UCEC, and LIHC. Whereas lower *PCSK9* expression in later stages was observed only in KIRC, KIRP, and LUAD.

### 3.2 *PCSK9* genetic alterations (mutations and DNA methylation) in various cancers

**Figure 2:**
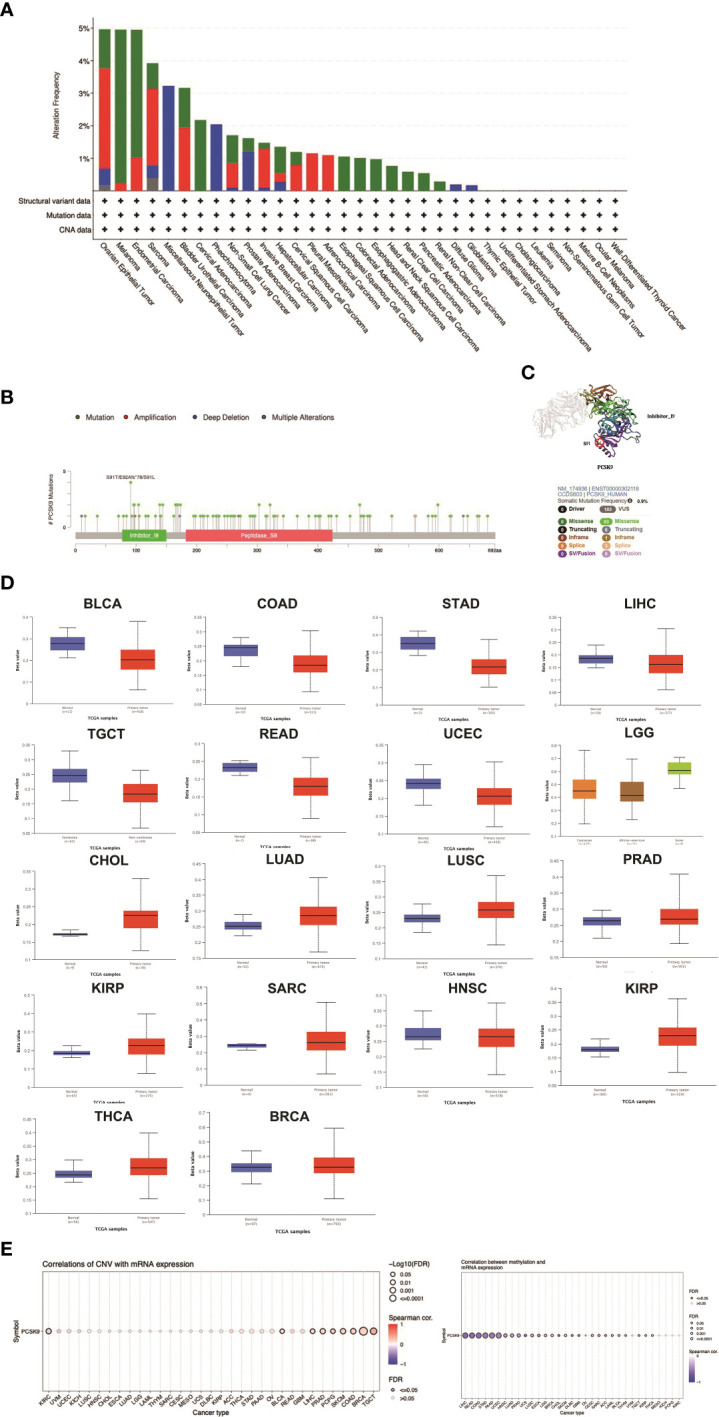
DNA methylation and mutation features of PCSK9 across cancer types. **(A)** The alteration frequency with different types of mutations was examined using the cBioPortal database. **(B, C)** The mutation site with the highest alteration frequency (E92Afs*78) in the 3D structure of PCSK9. **(D)** Promoter methylation level of PCSK9 across cancers. The results were obtained from the UALCAN database and GSCA database. **(E)** The correlation between PCSK9 expression and copy number variations (CNV) are shown from the GSCA database.

It has been widely acknowledged that genomic mutations are closely associated with tumorigenesis. To determine the genomic mutations of *PCSK9* in tumors, we reviewed the genetic alterations of the *PCSK9* gene in cancer patients using the cBioPortal database. Notably, patients with ovarian epithelial tumors had the highest frequency of *PCSK9* genetic alterations (5%), including amplification of copy numbers and deep deletions (**Figure 2A**). In addition, several cancer types (e.g., non-small cell lung cancer, cervical squamous cell carcinoma, ESCA, LIHC, SARC, BLCA, and BRCA) had *PCSK9* mutations (amplifications or deep deletions). Tumors with dominant *PCSK9* mutations included cervical adenocarcinoma, esophageal squamous cell carcinoma, esophagogastric adenocarcinoma, renal non-clear cell carcinoma, COAD, HNSC, KIRC, PAAD. Deep deletions were more common in various neuroepithelial tumors, PCPG, LGG, and GBM. These results revealed the highly heterogeneous inheritance and expression changes of PCSK9 in different types of cancer.

We next differentiated the distribution of mutations and collected the types, sites, and case number of genetic alterations of *PCSK*. As shown in **Figures 2B, C**, there were 93 missense and 6 truncation mutations in *PCSK9*. The site S91T/E92Afs*78/S91L, in the inhibitor_I9 domain, was confirmed to have the highest abundance of mutations. We future searched the cBioPortal website for PCSK9-related tumor gene mutations and identified six types of tumors with a total of eleven protein changes: Melanoma (D321N, E84K, G176E, E405K, S249G), Uterine Endometrioid Carcinoma (V79M), Colorectal Adenocarcinoma (R272L), Diffuse Large B-Cell Lymphoma (Q454H), Serous Ovarian Cancer (R93H, S5G), and Esophageal Adenocarcinoma (C526F). Subsequently, we conducted a search on the PolyPhen-2 website (http://genetics.bwh.harvard.edu/pph2/) and found that in five tumor types (Melanoma, Uterine Endometrioid Carcinoma, Diffuse Large B-Cell Lymphoma, Serous Ovarian Cancer, and Esophageal Adenocarcinoma), the protein changes were predicted to be either “probably damaging” or “possibly damaging.” However, the mutation in Colorectal Adenocarcinoma exhibited benign characteristics. Moreover, when we further correlated the prognosis of these six tumor types, we observed that three of them (Melanoma, Serous Ovarian Cancer, and Uterine Endometrioid Carcinoma) displayed worse prognoses, suggesting a potential association with inherent mutations (**Supplementary Table 1**).

DNA methylation is an epigenetic modification that can alter gene expression. The alteration of DNA methylation may be an essential factor in tumorigenesis. Through the UALCAN database, we explored *PCSK9* methylation between tumors and normal tissues. The results showed that the methylation of *PCSK9* was upregulated in various tumors, including BRCA, CHOL, HNSC, KIRC, KIRP, LGG, LUAD, LUSC, PRAD, SARC, and THCA (**Figure 2D**). Accordingly, these results indicated that *PCSK9* may mediate tumorigenesis by regulating DNA damage or methylation status in human cancers. Furthermore, similar results were concluded from GSCALite data, which revealed a relatively high mutation frequency in diverse types of cancer. We also investigated the frequency of CNV changes of *PCSK9*. The results showed that tumors, including TGCT, BRCA, COAD, SKCM, PCPG, PRAD, LIHC, BLCA and KIRC, were significantly correlated with the CNV changes (**Figure 2E**).

### 3.3 Multifaceted prognostic value of *PCSK9* across cancers

**Figure 3:**
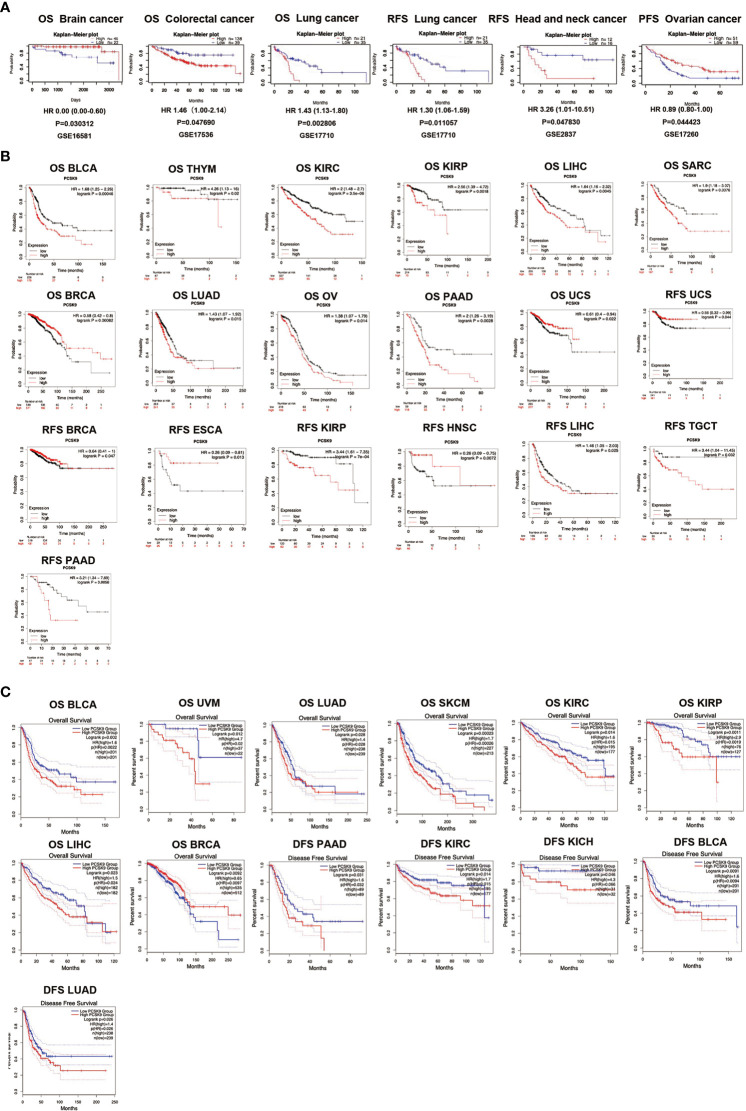
**(A)** Survival analyses of *PCSK9* expression across cancers (based on PrognoScan). OS (n = 67) in brain cancer cohort GSE16581. OS (n = 177) in colorectal cancer cohort GSE17536. OS (n = 56) in lung cancer cohort GSE17710. RFS (n = 56) in lung cancer cohort GSE17710. RFS (n = 28) in head and neck cancer cohort GSE2837. PFS (n = 110) in ovarian cancer cohort GSE17260. OS, overall survival; RFS, relapse free survival; PFS, progression free survival. **(B)** Kaplan-Meier survival curves of survival comparing high and low expression of *PCSK9* in the Kaplan-Meier Plotter database. Overall survival differences between groups in BLCA, THYM, KIRC, KIRP, LIHC, SARC, BRCA, LUAD, OV, PAAD, PCPG, and UCS. (M–T) Relapse-free interval difference between groups in UCS, BRCA, ESCA, KIRP, HNSC, LIHC, TGCT, and PAAD. **(C)** Kaplan-Meier survival curves of survival comparing high and low expression of *PCSK9* in the GEPIA database. (A–H) Overall survival differences between groups in BLCA, UVM, LUAD, SKCM, KIRC, KIRP, LIHC, and BRCA. (I–M) Disease-free interval difference between groups in PAAD, KIRC, KICH, BLCA, and LUAD.

The PrognoScan database was used to explore the relationship between *PCSK9* expression and prognosis in each cancer, results are summarized in **Figure 3**. Notably, *PCSK9* expression was significantly associated with five cancer types: brain, colorectal, lung, head and neck, and ovarian cancer (**Figure 3A**). Among them, *PCSK9* showed a detrimental relationship in three cancer types, including colorectal cancer [OS: total = 177, HR = 1.46, Cox *p* = 0.048], lung cancer [OS: total = 56, HR = 1.43 Cox *p* = 0.028], lung cancer [RFS: total = 56, HR = 1.30, Cox *p* = 0.011] and head and neck cancer [RFS: total = 28, HR = 3.26, Cox *p* = 0.048]. However, *PCSK9* exhibited a protective effect in two other cancer types, brain cancer [OS: total = 67, HR = 0.01, Cox *p* = 0.030] and ovarian cancer [PFS (progression free survival): total = 110, HR = 0.89, Cox *p* = 0.044] (**Figure 3A**).

To evaluate the relationship between PCSK9 protein expression levels and the prognosis of tumor patients, the Kaplan-Meier Plotter database was used to study the PCSK9 protein expression level in 21 cancer tissues (**Figure 3B**). In some cancer tissues, such as BLCA (OS, HR = 1.68, *p* = 0.0005), THYM (OS, HR = 4.26, *p* = 0.02), KIRC (OS, HR = 2.56, *p* = 0.002), LIHC (OS, HR = 1.64, *p* = 0.005), SARC (OS, HR = 1.9, *p* = 0.008), LUAD (OS, HR = 1.43, *p* = 0.015), OV (OS, HR = 1.38, *p* = 0.014), PAAD (OS, HR = 2.00, *p* = 0.003), KIRP (RFS, HR = 3.44, *p* = 0.001), LIHC (RFS, HR = 1.46, *p* = 0.025), TGCT (RFS, HR = 3.44, *p* = 0.032), and PAAD (RFS, HR = 3.21, *p* = 0.006), high expression of PCSK9 correlated with poor OS and RFS. Other cancers exhibited a protective role for PCSK9 with low expression, such as BRCA (OS, HR = 0.58, *p* = 0.001; RFS, HR = 0.64, *p* = 0.047), UCS (OS, HR = 0.61, *p* = 0.022; RFS, HR = 0.56, *p* = 0.044), ESCA (RFS, HR = 0.26, *p* = 0.013), and HNSC (RFS, HR = 0.26, *p* = 0.007).

Similar work was also performed using the GEPIA database. High expression of PCSK9 was associated with a poor OS prognosis in BLCA (*p* = 0.002), UVM (*p* = 0.012), LUAD (*p* = 0.028), SKCM (*p* = 0.0002), KIRC (*p* = 0.014), KIRP (*p* = 0.001), LIHC (*p* = 0.023), and BRCA (*p* = 0.009) (**Figure 3C**). The data showed that high expression of PCSK9 was associated with an adverse DFS prognosis in PAAD (*p* = 0.031), KIRC (*p* = 0.014), KICH (*p* = 0.046), BLCA (*p* = 0.009), and LUAD (*p* = 0.026) (**Figure 3C**).

**Figure 4:**
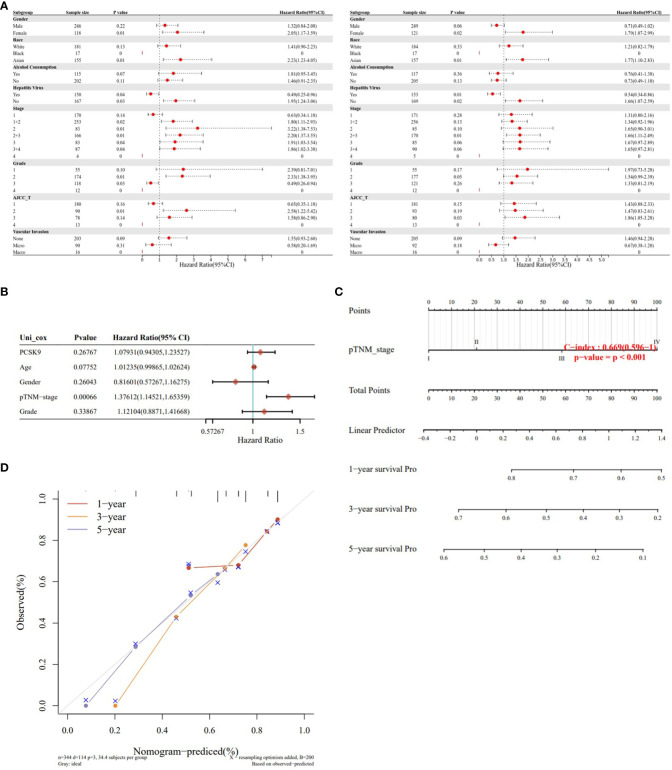
Survival analysis of PCSK9 expression in different clinicopathologic features in hepatocellular carcinoma. **(A)** Correlation of PCSK9 mRNA expression with OS in LIHC. **(B)** Correlation of PCSK9 mRNA expression with PFS in LIHC. OS, overall survival; PFS, progression free survival. **(C, D)** Development of a nomogram prediction model using PCSK9 expression levels and clinicopathological parameters for survival prediction.

To verify the correlation between PCSK9 expression and multiple clinicopathological characteristics, we used LIHC as an example. The results indicated that PCSK9 was associated with a detrimental prognosis with five patient or tumor characteristics: female (OS: *p* = 0.01; PFS: *p* = 0.02), Asian race [OS: *p* = 0.01; PFS: *p =* 0.01], no hepatitis virus infection [OS: *p* = 0.03; PFS: *p* = 0.02], pathology stage 2 [OS: *p* = 0.01] and stage 3 [OS: *p* = 0.04], grade 2 [OS: *p* = 0.01], and AJCC stage 2 (OS: *p* = 0.01) and stage 3 (PFS: *p* = 0.03) (**Figure 4A**). A nomogram prediction model was generated by integrating the above clinicopathological parameters and *PCSK9* expression levels. The calibration curve is further evaluated to show that the predictions made by nomogram is in considerable agreement with the actual survival (**Figures 4B–D**).

### 3.4 Correlation of PCSK9 expression and stemness

**Figure 5:**
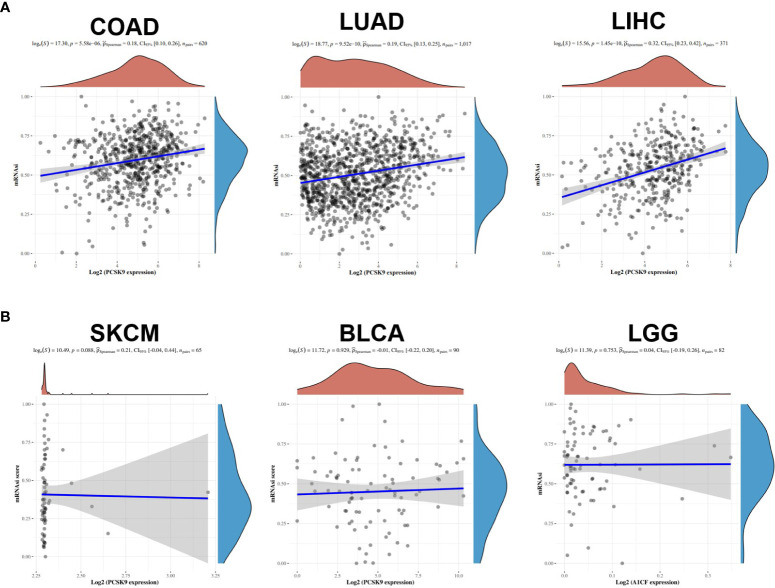
Associations of stemness indices with the PCSK9 expression in various cancers. **(A)** Positive correlations between mRNAsi and PCSK9 in COAD, LUAD and LIHC (p < 0.05). **(B)** No correlation between mRNAsi and PCSK9 expression in SKCM, BLCA and LGG (p > 0.05).

Cancer progression involves the gradual loss of differentiated phenotypes and stemness features. Our correlation analysis revealed that the expression of *PCSK9* was positively correlated with the mRNAsi in COAD, LUAD and LIHC. As for tumors such as SKCM, BLCA and LGG, there is no correlation between the characteristics of stemness and the expression of *PCSK9* (**Figures 5A, B**).

### 3.5 KEGG, GO, and GVSA analysis of PCSK9

**Figure 6:**
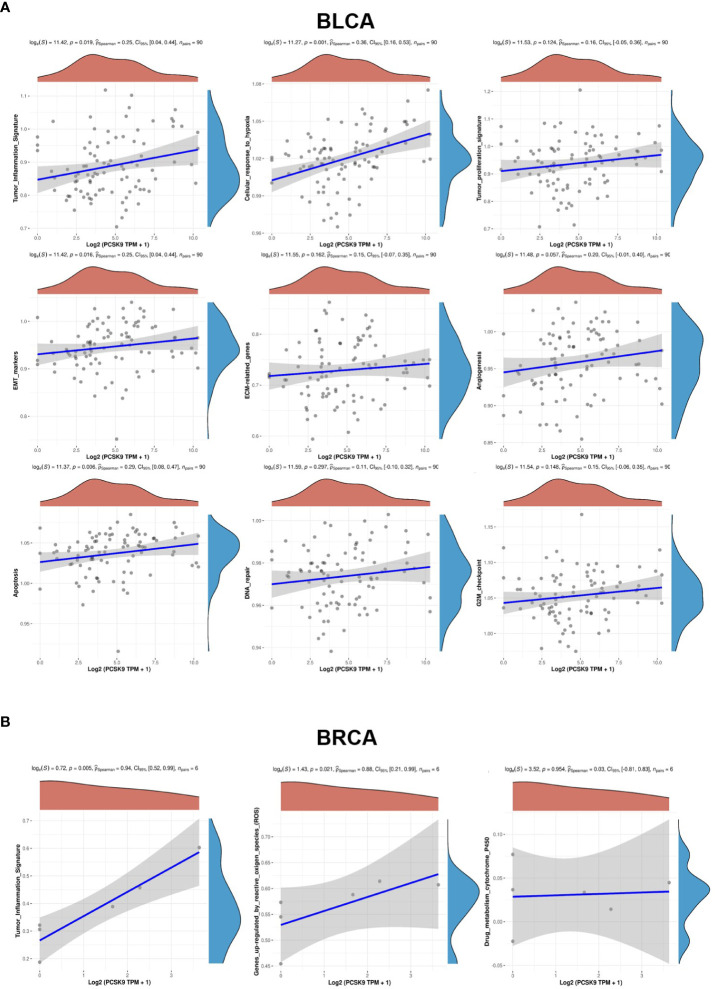
GVSA analysis of PCSK9 in various tumors. **(A)** BLCA, **(B)** BRCA.

**Figure 7:**
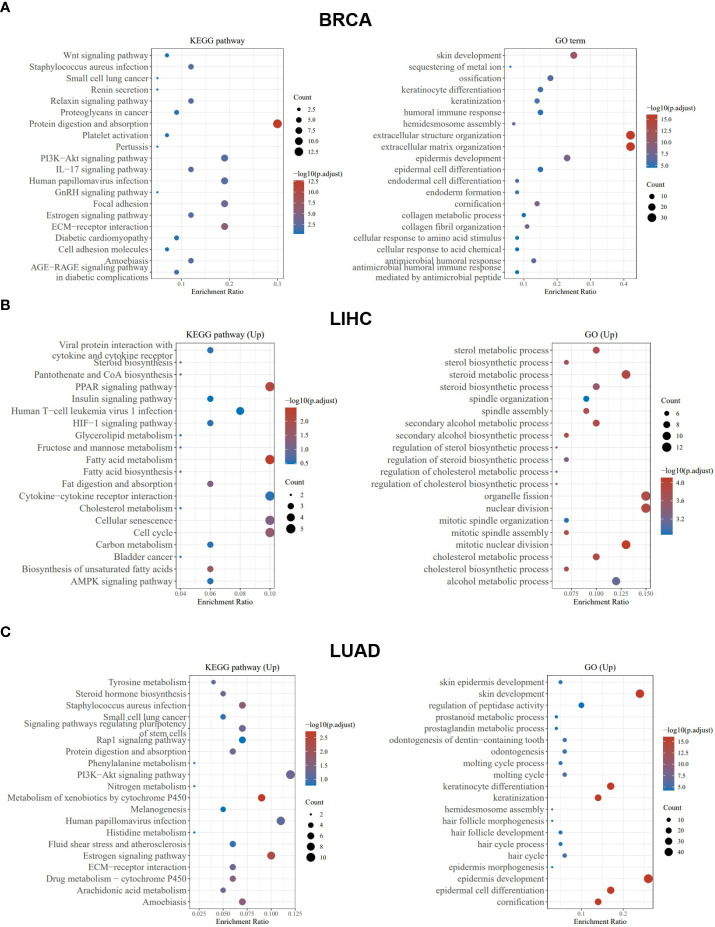
PCSK9-related KEGG pathway analysis and GO enrichment analysis. **(A–C)** KEGG pathway analysis and GO enrichment analysis of biological processes based on the PCSK9-interacted and PCSK9-correlated genes in BRCA, LIHC and LUAD.

Taking BLCA as an example, we found that abnormal expression of *PCSK9* was associated with tumor invasion characteristics, cell hypoxia manifestations, and EMT-related markers (**Figure 6A**). In BRCA, *PCSK9* expression was involved in tumor inflammation signature, reactive oxygen species and transforming growth factor beta (TGF-β) (**Figure 6B**). The KEGG pathway analysis and GO enrichment analysis were performed to demonstrate the primary biological pathways and potential targets of major potential *PCSK9* mRNA. The results showed that PCSK9 positively regulated cellular adhesion, cholesterol and fatty acid metabolism, PI3K-Akt signaling pathway and immune-related functions in BRCA, LIHC and LUAD (**Figures 7A–C**).

### 3.6 Correlation of PCSK9 expression with immune infiltration and various subsets of immune cells

**Figure 8:**
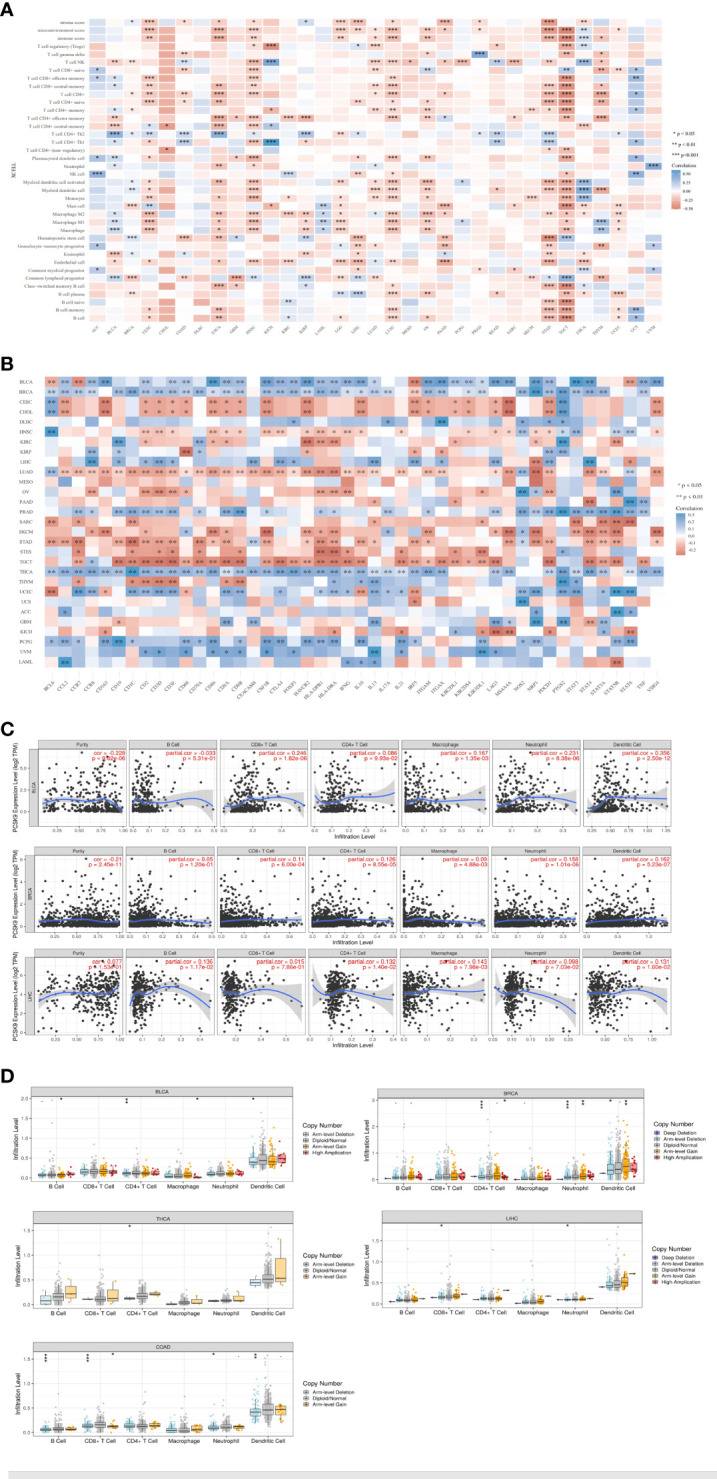
Correlation of *PCSK9* expression with immune infiltration and various subsets of immune cells. **(A)** Correlation of *PCSK9* expression with the levels of infiltrating immune cells based on xCell (*p < 0.05, **p < 0.01). **(B)** Correlation between *PCSK9* expression and immunosuppressive factors or immune stimulatory factors (*p < 0.05, **p < 0.01). **(C)** Correlation between *PCSK9* expression and infiltration scores of six immune infiltrates, including B cells, CD4^+^ T cells, CD8^+^ T cells, dendritic cells, macrophages, and neutrophils, in BRCA, BLCA, and LIHC. **(D)** The association between *PCSK9* copy number variations and immune infiltrates in LIHC, BLCA, BRCA, COAD, and THCA (i–v, *p < 0.05, **p < 0.01, ***p < 0.001).

Tumor-infiltrating lymphocytes are independent predictors of sentinel node status and cancer prognosis. The correlation between the expression levels of PCSK9 protein and immune cells in pan-cancer tissues were analyzed using the TIMER database. PCSK9 expression levels significantly correlated with immune cells (CD8+ T cells in 14 types of cancer, dendritic cells (DCs) in 11 types of cancer, and macrophages in 13 types of cancer). We further studied whether PCSK9 expression correlated with the infiltration of different immune cell subtypes using the xCell online tool. The results showed that PCSK9 expression was significantly correlated with subtypes of infiltrating immune cells in various tumors, including HNSC, TGCT, ESCA, COAD, STAD, LUSC, and LIHC (**Figure 8A**). CD8+ T cells, DCs, and M2 macrophages were the immune cell subtypes most positively associated with PCSK9 expression in these different cancers.

Immune checkpoint inhibitors (ICIs) are novel tumor immunotherapy agents that play an essential role in tumor immunotherapy. Subsequently, we analyzed the correlation between PCSK9 expression and immune checkpoint gene expression levels using the R software package in the SangerBox database. The correlations between 46 immune checkpoint genes and PCSK9 protein expression levels were calculated, and a significant relationship was found in many cancer types with many of the 46 genes, such as THCA (40 of 46), BRCA (37 of 46), LUAD (37 of 46), BLCA (35 of 46), and TGCT (33 of 46) (**Figure 8B**). Furthermore, the results showed that the expression of PCSK9 in BRCA, BLCA, PRAD, THCA, LIHC, PCPG, and UVM was negatively correlated with immune checkpoint genes. Furthermore, a positive correlation was found in CESC, CHOL, LUAD, STAD, and TGCT. Notably, these immune checkpoint genes contained a broad spectrum of immune regulators, including signaling chemokines, immune stimulators, immune inhibitors, and major histocompatibility complex molecules, such as those related to regulatory T (Treg) cells (chemokine receptor 8, *CCR8*; forkhead box protein p3, *FOXP3*; signal transducer and activator of transcription 5B, *STAT5B*), B cells (*CD19*), macrophages (*CD68*), interleukin-10 (*IL10*), Th17 (signal transducer and activator of transcription 3 (*STAT3*), neutrophils (integrin subunit alpha m, *ITGAM*), natural killer (NK) cells killer cell immunoglobulin-like receptor 2DL (*KIR2DL*), DCs (major histocompatibility complex class II DR beta 1 (*HLA-DPB1*); histocompatibility complex class II DR alpha (*-DRA*; integrin subunit alpha X(*ITGAX*), Th1 cells (interferon gamma (*IFNy)*; tumor necrosis factor (*TNF*), Th2 cells (signal transducer and activator of transcription 6 (*STAT6*; signal transducer and activator of transcription 5A (*STAT5A*), and exhausted T cells (programmed cell death protein 1 (*PDCD1*); *CTAL4*; hepatitis A virus cellular receptor 2 (*HAVCR2*). Additionally, our results showed a significant correlation between PCSK9 expression with *PDCD1* and *CTLA4*, which referred to T cell exhaustion and led to the loss of T cell function in patients with common chronic infections and cancer.

Immune infiltration plays a vital role in the TME. To evaluate the association between PCSK9 expression and immune infiltration, we further investigated the relationships between the PCSK9 expression and immune cells in three different cancers (BLCA, BRCA, and LIHC) from the TIMMER database (**Figure 8C**). The immune cells included B cells, CD8+ T cells, CD4+ T cells, macrophages, neutrophils, and DCs. In BLCA, the expression level of PCSK9 significant and positively correlated with CD4+ T cells (R = 0.126, *p* = 8.55E−05), CD8+ T cells (R = 0.11, *p* = 6.00E−04), macrophages (R = 0.09, *p* = 4.88E−03), neutrophils (R = 0.158, *p* = 1.01E−06), and DCs (R = 0.162, *p* = 5.23E−07). In BLCA, the correlation between the expression level of PCSK9 with immune cells was significant, including CD8+ T cells (R = 0.246, *p* = 1.82E−06), CD4+ T cells (R = 0.086, *p* = 9.93E−02), macrophages (R = 0.167, *p* = 1.35E−03), neutrophils (R = 0.231, *p* = 8.38E−06), and DCs (R = 0.356, *p* = 2.50E−12). In LIHC, we also found a significant correlation of PCSK9 with B cells (R = 0.136, *p* = 1.17E−02), CD4+ T cells (R = 0.132, *p* = 1.40E−02), macrophages (R = 0.143, *p* = 7.98E−03), neutrophils (R = 0.098, *p* = 7.03E−02), and DCs (R = 0.131, *p* = 1.60E−02). There was no correlation between PCSK9 and B cells in BRCA or BLCA (*p* > 0.05), or LIHC with CD8+ T cell (*p* > 0.05). In addition, PCSK9 expression strongly correlated with tumor-associated macrophages and DCs in these three cancers. These findings strongly suggested that PCSK9 affected the immune microenvironment by interacting with immune cell infiltration in various cancers.

Considering the heightened sensitivity of gamma delta T cells, particularly gamma 9 delta 2 T cells, towards alterations in the cholesterol pathway, it would be significant to include these cells as a specific control in the immune infiltration analysis. Thus, we further evaluated the immune cell infiltration scores associated with tumor samples obtained from the TCGA database. By utilizing the “corr.test” function in R software, we ultimately identified a significant correlation between PCSK9 expression and gamma delta T cell immune infiltration scores. Specifically, we observed a significant association between PCSK9 expression and gamma delta T cell infiltration in five types of tumors (TGCT, COAD, PRAD, LUSC and LIHC). These results indicated incorporating gamma delta T cells into the analysis would provide additional insights into the potential impact of PCSK9 and its role in modulating immune responses within the tumor microenvironment (**Supplementary Figure 1**).

To further unravel the potential predictive value of *PCSK9* gene alterations for ICI treatment, we then investigated the relationship between *PCSK9* alterations and six common immune infiltrates (B cells, CD4+ T cells, CD8+ T cells, macrophages, neutrophils, and DCs) across multiple cancer types. We demonstrated that *PCSK9* is frequently altered across different cancer types. Furthermore, we investigated the relationship between the *PCSK9* CNV and immune infiltration in various cancers (**Figure 8D** i–v). For six types of immune cells, we verified a correlation of the *PCSK9* CNV with immune infiltration. CD8+ and CD4+ T cells were associated with *PCSK9* deletions in LIHC, BLCA, BRCA, and THCA. Deletion of *PCSK9* was significantly related to infiltration of CD4+ T cells (*p* = 0.001), neutrophils (*p* = 0.001), and DCs (*p* = 0.05) in BRCA. These results suggested a possible mechanism by which immune cells may be affected by PCSK9 in the TME, which may help direct future immunotherapy treatments.

### 3.7 Correlation of PCSK9 expression and clinical chemotherapies

**Figure 9:**
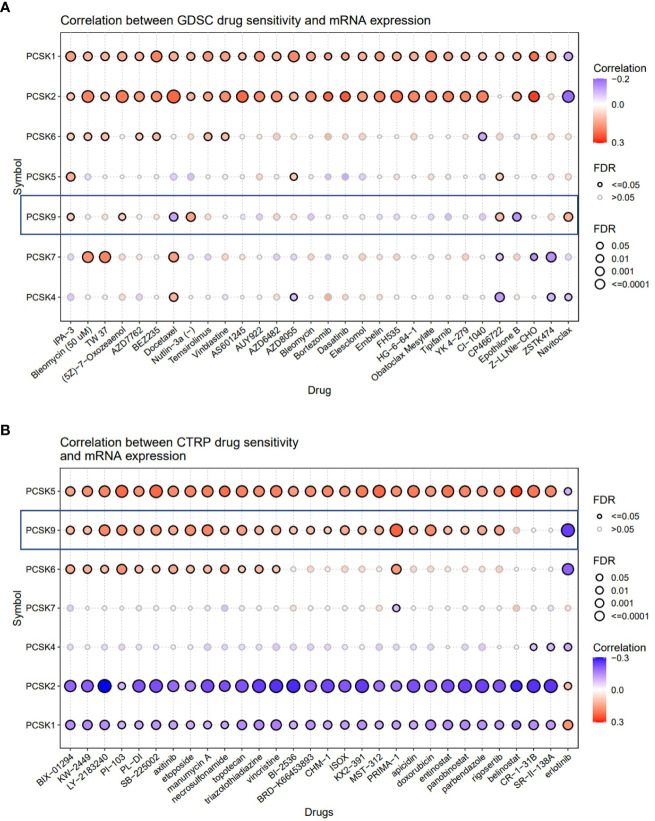
Correlation analysis between PCSK9 expression and drug sensitivity. **(A)** Correlation between PCSK9 and sensitivity of the top 10 anticancer drugs in GDSC database. **(B)** Difference of drug sensitivity between PCSK9-related expression groups in CTRP database.

GDSC showed that high expression of *PCSK9* could make cancer more sensitive to IPA-3 (target PAK1), (5Z)-7-Oxozeaenol (target TAK1), Nutlin-3a (target MDM2), Navitoclax (target BCL2) and resistant to Docetaxel (target microtubule stabilizer), Epothilone B (target microtubule stabilizer), OSU-03012 (target PDK1) (**Figure 9A**). While in CTRP database, IC50 of QW-BI-011, CCT036477, CIL70, PRIMA-1, PRIMA-1-Met, teniposide, ML210, BRD-K92856060, BRD-K26531177 and avrainvillamide showed top 10 significant positive correlations with the expression level of *PCSK9* (**Figure 9B**). Collectively, these results may provide new ideas for developing potential drugs relating to the expression of *PCSK9* for treating cancers.

### 3.8 Experimental verification of PCSK9 in neuroblastoma

**Figure 10:**
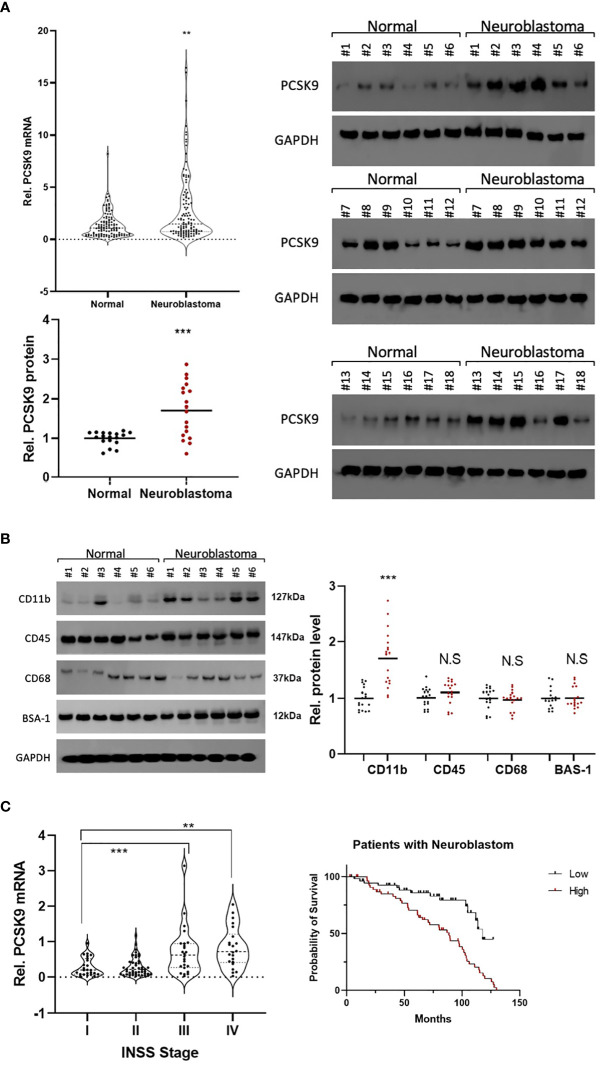
**(A)** Preliminary experimental verification of PCSK9 expression in neuroblastoma (NB). The mRNA and protein expression levels of PCSK9 in paired NB and adjacent non-tumor tissues by qRT-PCR (n=18; **Figure 1A**) and western blotting (n=18; **Figures 1B, C**) **p < 0.01, ***p < 0.001; **(B)** The association between NK cell-relevant immune checkpoints (CD11b, CD45, CD68) and PCSK9 expression in NB was detected by western blot in 18 NB tissue samples (***p < 0.001). **(C)** The association between PCSK9 expression in different clinical stages and survival analysis of patients. WHO stage III and IV showed highly expressed PCSK9 that correlated with poor progression-free survival (PFS, HR=1.51, 95% CI 1.25–1.71, p < 0.05).

To confirm PCSK9 expression in NB, we measured mRNA and protein levels of PCSK9 in paired NB and adjacent non-tumor tissues using qRT-PCR (n = 18; **Figure 10A**) and western blotting (n = 18; **Figure 10A**). Our findings revealed that PCSK9 expression in tumor tissues was significantly higher at both the transcriptional and protein levels than in adjacent normal tissues (*p* < 0.001).

Immune cells play an essential role in tumor immune tolerance. Immunotherapy (such as disialoganglioside GD2) has been incorporated into first-line treatment regimens of relapsed NB to significantly improve patient outcomes. Remarkably, immune cells, such as NK cells and macrophages, are thought to be the main effectors of anti-GD2 antibody potency in NB tumors. Thus, to investigate the role of immune cells in the NB TME, we used western blotting to measure the levels of CD11b, CD45, and CD68 expression in NB tissues (**Figure 10B**). The results showed that CD11b (PDC#2, PDC#4, and PDC#5) was positive for immune infiltration of NB, which represented the potential involvement of NK cells in immune infiltration (**Figure 10B**).

Furthermore, we investigated the PCSK9 prognostic value in NB patients. Upregulated PCSK9 was associated with a lower PFS in the NB cohort, which was consistent with our database survival analysis (HR = 1.51, **Figure 10C**). Our findings showed that PCSK9 was negatively associated with NK cell infiltration in NB, implying a potential role of PCSK9 modulation in the NB microenvironment.

## 4 Discussion

Pan-cancer evaluations are increasingly being used to uncover functional genes, particularly those with immunological roles in oncology. To better understand the commonalities and heterogeneities during the fundamental biological processes of distinct malignancies, pan-cancer approaches have been employed. The data from such studies is beneficial for developing novel strategies of cancer prevention and treatment targets (26).

Reports of PCSK9 involvement in the management of dysregulated cholesterol levels and atherosclerotic cardiovascular diseases are well established. For example, PCSK9 inhibition during coronary artery disease is recognized as an effective therapeutic strategy. Additionally, several studies on cholesterol regulation in tumor sites have been proposed to be due to the corresponding association between PCSK9 and oncogenesis (27, 28). Therefore, PCSK9 regulation in physiological processes such as cancer cell death and cell proliferation is in addition to its role in cholesterol homeostasis (10). The finding that PCSK9 and PD-1 might collaborate to enhance T cell immunological tolerance suggests a potential role in the immunotherapy of tumors. However, the function of PCSK9 in human cancers has not been determined, and it is not yet known if PCSK9 could have a significant impact on the immunological crosstalk associated with cancer and, in turn, affect the prognosis of different cancer types. Our novel findings are discussed below.

First, we conducted extensive data searches from TIMER to investigate the levels of *PCSK9* expression in diverse tumor types. The results revealed dysregulated *PCSK9* expression in different cancers. *PCSK9* expression was shown to be considerably higher in the following tissues: BRCA, CESC, CHOL, COAD, ESCA, HNSC, LIHC, READ, SKCM, STAD, THCA, and ECEC. Low levels of *PCSK9* were expressed in malignancies of the brain, kidney, lung, and prostate. Our results further detailed that tumor with substantial *PCSK9* expression may express different levels at various stages and subtypes, based on the GEPIA and UALCAN databases. DNA methylation has recently been demonstrated to serve significant regulatory functions in cancer development. In support of the positive relationship between DNA methylation and PCSK9 dysregulation, our study discovered that melanoma, non-small cell lung cancer, cervical squamous cell carcinoma, ESCA, LIHC, SARC, BLCA, and BRCA all had greater levels of *PCSK9* methylation. Taken together, aberrantly expressed *PCSK9* was related to cancer progression and cancer prognosis through modulation of *PCSK9* DNA methylation, an epigenetic hallmark of cancer.

Next, we visualized the prognostic landscape in human cancers using independent datasets from TCGA data in GEPIA and Kaplan-Meier Plotter. Our study indicated that *PCSK9* was a significant prognostic factor in various cancer types. However, an apparent heterogeneity was observed in different tumors regarding prognosis with some cancers exhibiting protective effects whilst others showed a pathogenic or an insignificant link. Concerning the heterogeneity, we further explored the correlation between *PCSK9* expression and several clinicopathological characteristics in different stages and grades of LIHC. The results showed that different factors could to some extent explain the heterogeneity of cancers that may have led to a protective or detrimental *PCSK9* prognostic relationship. These findings suggested that *PCSK9* may be a tumor molecular marker according to different forms of initiation and progression of LIHC.

Another significant discovery was the correlation between *PCSK9* expression and various infiltrating types of immune cells in the majority of cancer types. These cells, including Treg cells, DCs, macrophages, neutrophils, and tumor-infiltrating lymphocytes (B cells and T cells), are crucial for the development and spread of tumors (29, 30). For instance, Treg cells are thought to be suppressors of overactive immunological responses by producing CTLA4, IL-10, and TGF, which may allow tumor cells to evade the immune system (31, 32). Treg cells may also affect the proportion of CD4+ and CD8+ T cells and T cell differentiation (33). However, under the influence of the TME, Treg cells are immature and have a poor immune regulatory capacity, which could result in tumor immune escape. Studies have demonstrated the complex regulatory network of Treg cells in the TME, which makes Treg regulation in immunotherapy more challenging (34). Our research found a link between aberrant *PCSK9* expression and the presence of Tregs in a variety of malignancies (**Figure 10A**), which may help to explain the synergistic effects of PCSK9 and PD-1 in immunotherapy.

The degree of CD8+ T cell infiltration into a tumor is yet another process that has been correlated with improved prognosis (35, 36). CD8+ T cells serve crucial functions in the immune system of the tumor, not only by attracting other immune cells and increasing the immunological response, but also by increasing the effectiveness of cancer immunotherapy, and ultimately improving the prognosis of patients (37). Furthermore, our examination of the pan-cancer population highlighted a correlation between PCSK9 expression and CD8+ T cell subpopulations in LIHC and LUAD. These findings suggested that PCSK9 might be involved in a significant and essential role in the immune infiltration.

Immune checkpoints that are abnormally expressed in the immune cell membrane or that act through receptors on cell membranes have the potential to be modulated by an oncogene (38). In this study, we gathered expression information on over 40 common immune checkpoint genes and investigated the connection between PCSK9 and these immune checkpoint genes. Using LIHC as an example, upregulated PCSK9 expression was positively linked with immunosuppressive checkpoint genes such as *PDCD1* (encoding PD-1), *CTLA4*, and *LAG3*, which are primarily expressed in exhausted T cells and consequently influence the prognosis of LIHC (39). A correlation of co-inhibition of *PCSK9* and *PDCD1* or *CTLA4* immune checkpoints could explain the enhanced effect of combining two or more ICIs in the 2nd generation immune therapy strategy (37). *FOXP3* and *CCR8* strongly correspond with *PCSK9* expression in LIHC, indicating that they may be involved in adaptive immune responses such as Treg cell-mediated immune response control (40). Next, we chose LUAD as another example, as we previously revealed that *PCSK9* was downregulated in LUAD patients, and associated with a poorer prognosis. In the immune checkpoint examination, a significant negative correlation existed between *PCSK9* and *CD163*, V-set and Ig domain-containing 4 (*VSIG4*), and membrane spanning 4-domains A4A (*MS4A4A*), which encode proteins exhibited on the surface of M2 macrophages, indicating the relationship with M2 macrophage polarization in the tumor microenvironment (41, 42). Furthermore, we also found a significant negative relationship between *PCSK9* overexpression and *NOS2*, which encodes a protein found on the surface of M1 macrophages. Collectively, these findings elucidated a possible stimulus function of M2 polarization and inflammation when considered jointly. Above all, these findings strongly suggest that *PCSK9* could be a future cancer immunotherapy target based on the interaction of immune cells in multiple cancer types.

The function of PCSK9 was not only to regulate cholesterol and prevent cardiovascular diseases, but also to potentiate the application of PCSK9 inhibitors in ischemia-reperfusion injury and enhance the synergistic anticancer effect of PD-1. All these highlighted the potential application prospect of PCSK9 inhibitors in clinic. Furthermore, recent research has unveiled an additional role of PCSK9 in the degradation of major histocompatibility complex I (MHC-I) receptors, exerting effects on the immune system and various physiological functions. This significant finding suggests a potential strategy of inhibiting PCSK9 to potentially augment T cell infiltration within tumors and enhance the response to immune checkpoint therapy. Overall, the discovery of PCSK9’s regulation of MHC I levels on cell surfaces represents a crucial breakthrough, providing valuable insights into immune infiltration within tumor microenvironments. Our study further analyzed the signal pathways that may performed in the tumorigenesis and revealed the sensitivity of PCSK9 correlated with anti-tumor drugs in tumor treatment and their corresponding targets, such as PAK1, BCL2 and MDM2. These results indicated the potential role of PCSK9 in chemosensitivity or resistance in different cancers. However, our study’s use of numerous datasets was not without its limitations. For example, past laboratory findings could not support a logical interpretation of the link between *PCSK9* expression and methyltransferase gene expression. Whilst useful conclusions could be drawn from our data based on numerous pan-cancer patient datasets, proof of concept clinical trials are needed to validate them. Specifically, the execution of functional tests and mechanistic investigations in *in vivo* and *in vitro* research, as well as clinical trials, are therefore needed for further analysis. Finally, even though we were able to show that *PCSK9* expression was associated with tumor immune cell infiltration and patient survival, we were not certain that *PCSK9* affected clinical survival through the immunological system; this requires further clinical trial verification.

Recently, the association between *PCSK9* and extracellular vesicle-derived miRNA has been studied in the context of cardiovascular disease (43). Studies have found that extracellular vesicles (EVs) can be induced by *PCSK9* and hence transport miRNAs to target cells, where the miRNAs can further modulate the expression of target genes, including LDLR and TLR4 (44). The interaction between *PCSK9* and EVs in tumorigenesis was interesting, but due to limited research, our pan-cancer analysis cannot reveal this point. Further study is of great significance to fully understand the specific mechanisms of the interaction between EVs and miRNAs to regulate the expression of *PCSK9* and its influence on tumor mechanisms. Interestingly, emerging data show that the circulating concentration of *PCSK9* in women is higher than that in men, which indicates that the potential roles of *PCSK9* may be different according to gender (45, 46). However, these researches of sex-related *PCSK9* mainly focused on cardiovascular diseases. As we mentioned that some studies have found that higher levels of *PCSK9* are associated with increased risk of colorectal cancer and liver cancer in both men and women. And some breast cancer patients showed that women with higher levels of *PCSK9* had a higher risk of developing metastatic disease. Hence, about the relationship between sex-related *PCSK9* and tumor, it needs to be further explained. Since the expression of *PCSK9* is sex-related, research is needed to explore if there are differences in the association of *PCSK9* and cancer risk between men and women. Although our pan-cancer analysis did not include related gender factors, the relationship between the abnormal expression of *PCSK9* related to gender and the prognosis, immune infiltration and treatment of tumor deserves further consideration. Since the current *PCSK9* inhibitors have been used in clinic, and the incidence of subsequent related tumors in these patients can be compared in a prospective study to help prove the potential of *PCSK9* in tumor immunotherapy.

To the best of our knowledge, this is the first report based on data mining and in-depth bioinformation analysis on the comprehensive molecular characteristics of *PCSK9* across diverse cancer types. Furthermore, we found a strong correlation between *PCSK9* expression and an immune checkpoint marker and immune cell infiltration levels. Finally, our observation that aberrant PCSK9 expression was associated with a poorer prognosis and immunity in neuroblastoma is consistent with previous pan-cancer findings. Further investigations and clinical trials are warranted to validate the utility of PCSK9 as a reliable biomarker and explore its potential role in guiding immunotherapeutic interventions for cancer treatment.

## Data availability statement

The data used to support the findings of this study can be available in the article. All the raw data of this study can be directed to the corresponding author upon request.

## Ethics statement

The studies involving humans were reviewed and approved by the Ethics Committee of the Children's Hospital of Suzhou University (No. 20170606013). The studies were conducted in accordance with the local legislation and institutional requirements. Written informed consent for participation in this study was provided by the participants or the participants' legal guardians/next of kin.

## Author contributions

Conception and design were performed by ZW. There was no administrative support. Study materials or patients were provided by SH. Data was collected and assembled by CS and GZ. Data analysis and interpretation were carried out by CHS, JL, ZM, and RL. The manuscript was written by all authors. The final version of the manuscript was approved by all authors.

## Supplementary material

Supplementary Table 1 PolyPhen-2 predicts the impact of mutations of PCSK9 on protein function.

Supplementary Figure 1 The correlation between PCSK9 expression and gamma delta T cell immune infiltration scores.
