# Supplementary figures and images for: Identification and validation of PCSK9 as a prognostic and immune-related influencing factor in tumorigenesis: a pan-cancer analysis
Source: Front Oncol. 2023 Oct 4;13:1134063. doi: 10.3389/fonc.2023.1134063 (PMC10584329; doi:10.3389/fonc.2023.1134063)

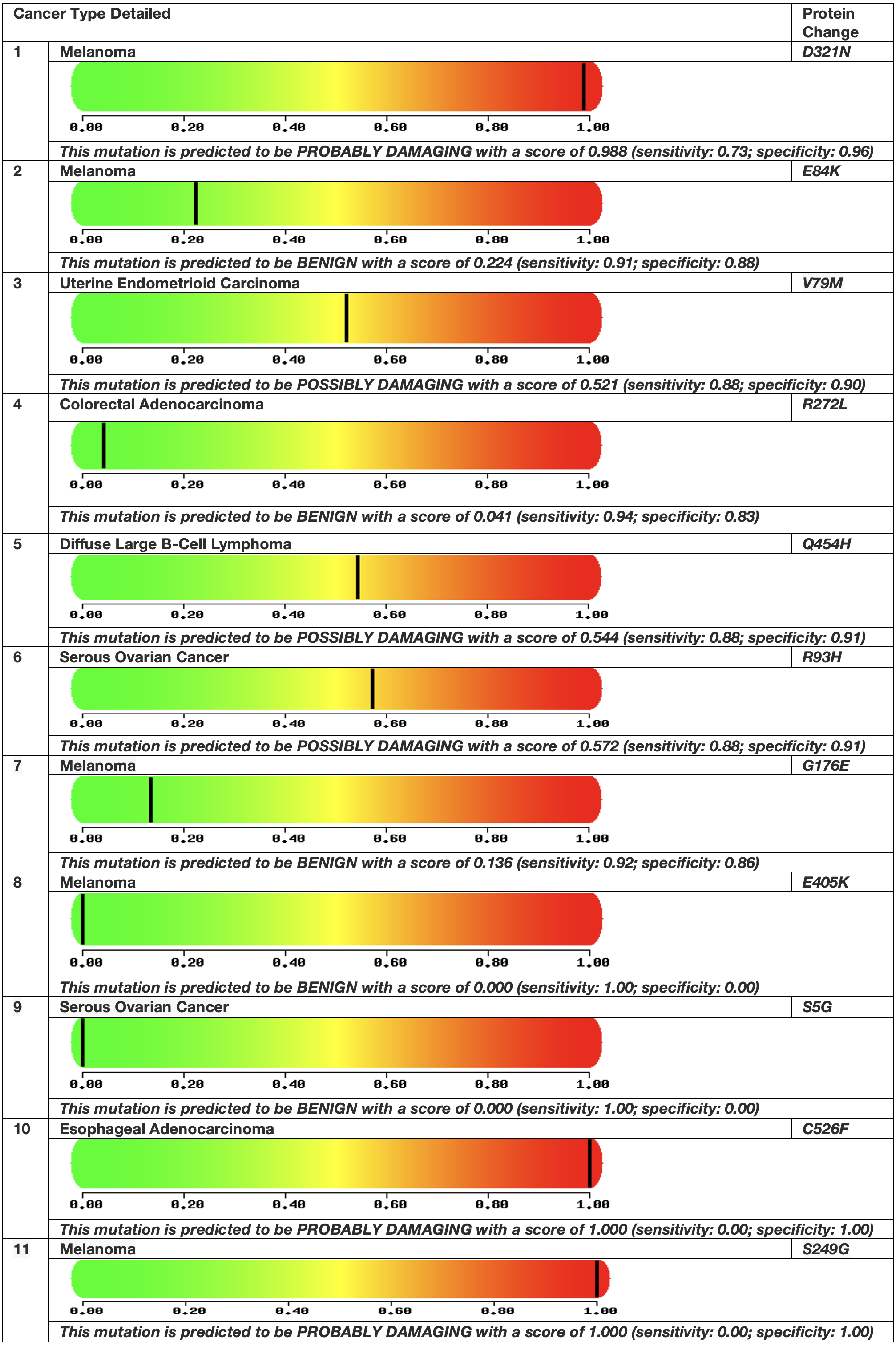

Supplement: Supplementary Table 1 — PolyPhen-2 predicts the impact of mutations of PCSK9 on protein function. [file Image_1.png]

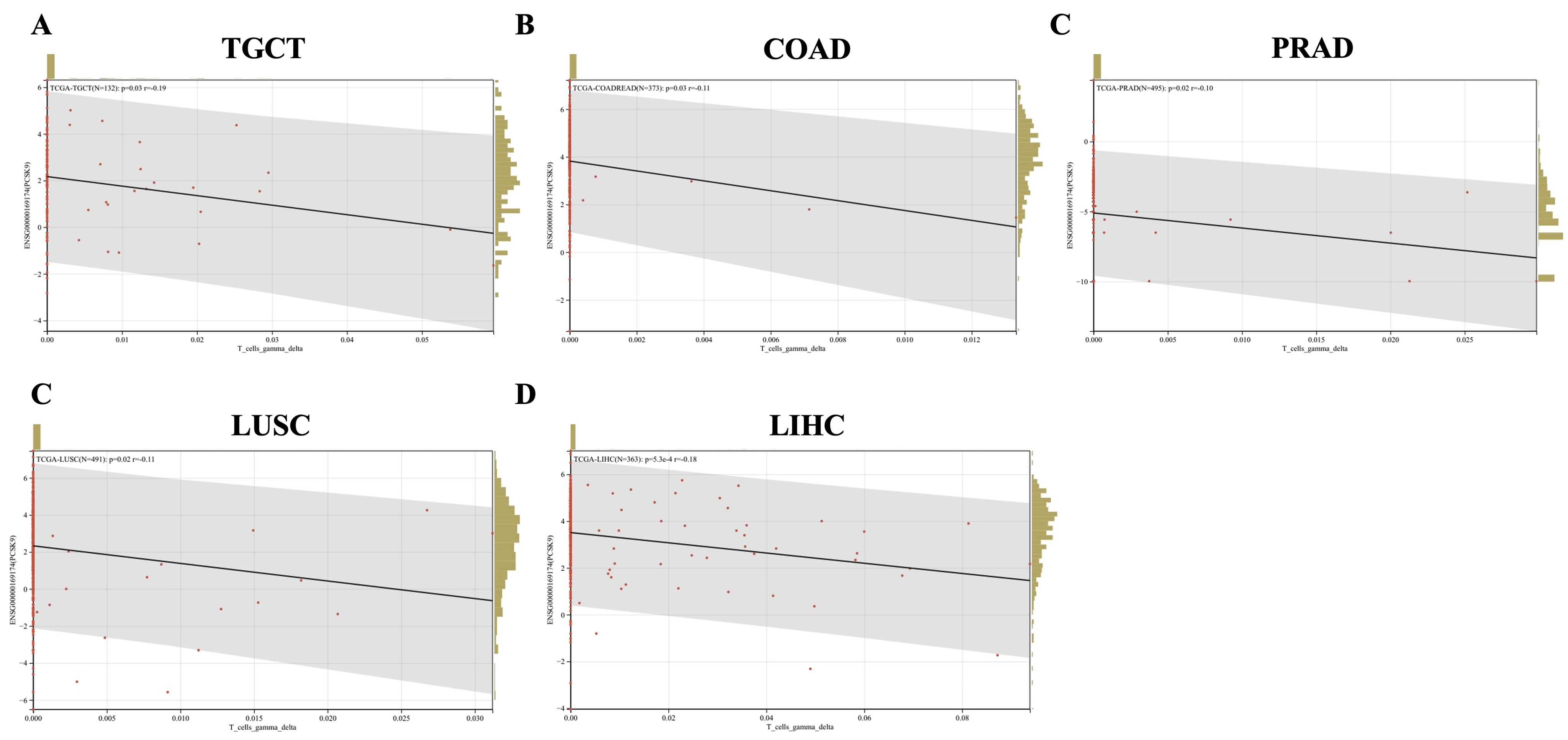

Supplement: Supplementary Figure 1 — The correlation between PCSK9 expression and gamma delta T cell immune infiltration scores. [file Image_2.png]
